# New Strategies for the Reduction of Uremic Toxins: How Much More We Know

**DOI:** 10.3390/toxins13120837

**Published:** 2021-11-24

**Authors:** Maria Teresa Rocchetti

**Affiliations:** Department of Clinical and Experimental Medicine, University of Foggia, 71122 Foggia, Italy; mariateresa.rocchetti@unifg.it

The importance of uremic toxin (UTx) removal in chronic kidney disease (CKD) is an emerging topic in the literature, widely recognized over time as a strategy to slow-down the disease progression towards end-stage renal disease and, consequentely, the occurence of deleterious effects on cardiovascular (CV) system [1,2], brain [3], lungs, and gut [4]. UTx start to accumulate in the blood of CKD patients, especially in advanced stages, where kidneys are no longer able to manage UTs, activating inflammation, oxidative reactions, and inducing profibrotic effects, all probable causes of kidney damage progression [5]. A bidirectional relationship between the gut microbiota and kidney, in both physiological and disease conditions, has been consolidated by literature data, demonstrating a status of dysbiosis of the CKD intestinal microbiota which overproduces proteolytic microbial derivatives, contributing to disease progression [6]. Among these microbiota-derived uremic toxins, indoxyl sulfate (IS) and para-cresyl sulfate (PCS) are being recognized as nontraditional risk factors of cardiovascular disorder in CKD [1,2,5,6]. Due to their high binding affinity to serum albumin, IS and PCS are not sufficiently removed by hemodialysis (HD) treatment, and therefore therapeutic strategies reducing their production and increasing their removal are expected to be advantageous. Moreover, HD treatment can be largely inefficient in the case of high molecular weight toxins. In this Special Issue, Yamamoto et al. [7] demonstrated that the protein binding properties of UTx in vitro were pH-dependent, suggesting the modification of blood pH, while passing through the dialyzer, as s future potential strategy to weaken UTx protein bonds and increase their removal with HD treatment.

In a context other than that of CKD uremic patients, HD is also recommended as the first-line treatment of acute hyperammonemia in neonates affected by inborn errors of metabolism in order to avoid brain toxicity. To detoxify ammonia, Eloot et al. [8] developed an algorithm useful for clinicians to conceptualize a specific protocol to treat acute neonatal hyperammonemia based on a patient’s characteristics.

Improvements in dialysis membranes and techniques to remove medium–high molecular weight uremic toxins without a significant albumin loss were shown by expanded hemodialysis (HDx), a dialysis modality in which diffusion and convection are combined inside a hollow-fiber dialyzer containing a medium-cut-off (MCO) high-retention-onset membrane [9]. In contrast, vitamin E-bonded membranes showed no benefit in decreasing by-products of oxidative stress and inflammation in dialysis patients lacking glutathione transferase M1 enzyme activity [10]. A paradigm shift from conventional dialysis therapies was represented by binding competitor-augmented hemodialysis. In this method, the binding competitor (i.e., ibuprofen, short-chain fatty acids, tryptophan) was infused upstream of a dialyzer into an extracorporeal circuit, to increase the free PBUTs fraction, competing with it for their albumin binding sites [11]. Although binding competition during dialysis showed high potential for PBUT removal, more research is required before it can be used in clinical practice. Even in peritoneal dialysis, new solutions containing L-carnitine and xylitol are being developed to preserve the peritoneal membrane integrity, in this way improving the removal of uremic solutes [12].

Magnani and Atti [13] presented the state of the art for blood purification strategies, showing that adsorption-based extracorporeal techniques, in particular hemodiafiltration with endogenous infusion (HFR) and hemoperfusion (HP), integrated directly in the current HD systems, adsorbed a significant amount of middle molecular weight molecules and PBTUs. However, the authors concluded by emphasizing that blood purification strategies used alone are not sufficient and future directions should include a synergic approach by reducing PBUTs production in the upstream and increasing their clearance in the downstream [13,14]. The same concept was supported by Eric and coworkers [15], who, after a systematic review on the effects of the use of a medium cut-off membrane (MCO) and dietary fiber on the serum level of PBUTs and inflammatory markers in HD patients, presented a protocol for an interventional trial using a combination of the two, namely MCO membrane dialysis and fiber supplementation.

Despite the progress in dialysis treatment of uremic patients, non-extracorporeal therapies, such as medicament (intestinal chelators and activated charcoal adsorbent [16]), nutritional therapy (i.e., low protein diet [17] or very low protein diet [18,19]) and supplement therapies (prebiotic, probiotic, synbiotic [20,21]) alone, in combination with each other [22] or in combinations with dialysis [14], are increasing, in order to reduce UTx production, improve kidney function and prevent CV complications. Peripheral vascular disease (PVD) developed by CKD patients could aggravate vascular complications and increase mortality risk [23]. Wu and colleagues [23] reviewed the role of UTs in the pathogenesis of PVD in CKD patients stressing the role of phosphorus and protein-bound uremic toxins and showing their promising role as a therapeutic target.

Laville et al. [16], in a narrative review, described the lack of effectiveness of phosphate binders sevelamer and, on the other hand, the efficacy of the sorbent AST-120 on the decrease in uremic toxin levels. Nutritional therapy, specifically a low protein intake, has been demonstrated to be effective in reducing the production of gut-derived UTs, in CKD patients [19], as well as in animals [17], and was suggested by Cupisti et al. in a combined approach with dialysis [18]. In their concept paper, the authors highlighted the importance of protecting the residual kidney function (RKF) which is crucial for uremic toxins excretion in stage 5 CKD patients. The strategy to decrease UTs suggested by the authors was represented by the combination of protein restricted diet with infrequent dialysis, useful for a “gradual, safe, and gentle beginning of dialysis” during the transition phase from conservative management to full HD treatment [18]. Accumulating evidence suggests the use of double approach as a successful strategy for the reduction of serum UTx, which consists of the combination of different methods acting upstream, such as at gut-microbiota level, and downstream, at a systemic level. Currently, in order to consolidate the effectiveness and applicability of this strategy in clinical outcome studies, clinicians need data derived from studies on extended treatment cycles with a larger number of patients.

In conclusion, it is of interest to note the many alternative strategies to reduce UTx serum levels that currently emerge as alternatives to traditional dialysis approaches. Nevertheless, for most of them, an improvement in uremic toxin concentration does not necessarily conform with a clinical benefit, and we should await confirmation in controlled hard outcome or quality of life studies in CKD patients before we can truly appreciate their relevance.
